# Clinical and genetic study of ABCB4 gene-related cholestatic liver disease in China: children and adults

**DOI:** 10.1186/s13023-024-03179-w

**Published:** 2024-04-12

**Authors:** Lili Cao, Xiuxin Ling, Jianguo Yan, Danni Feng, Yi Dong, Zhiqiang Xu, Fuchuan Wang, Shishu Zhu, Yinjie Gao, Zhenhua Cao, Min Zhang

**Affiliations:** 1 Department of Hepatology, Fifth Medical Center, PLA General Hospital, No.100, West Fourth Ring Road, Fengtai District, Beijing, 100039 China; 2 Grandomics Biosciences, Beijing, 100098 China

**Keywords:** MDR3/ABCB4 gene, Novel mutation, Liver pathology, Cholestatic liver disease

## Abstract

**Background:**

*ABCB4* gene-related cholestatic liver diseases have a wide spectrum of clinical and genetic variations. The correlation between genotype and clinical phenotype still unclear. This study retrospectively analyzed the clinical and pathological characteristics of 23 patients with *ABCB4* gene-related cholestatic liver diseases. Next-generation sequencing was used to identify the genetic causes.

**Results:**

The 23 included patients (15 children and 8 adults) were diagnosed as progressive familial intrahepatic cholestasis type 3 (PFIC3), drug-induced liver injury (DILI), cirrhosis cholestasis, cirrhosis, and mild liver fibrosis. Nineteen patients underwent liver pathological examination of the liver, exhibiting fibrosis, small bile duct hyperplasia, CK7(+), Cu(+), bile duct deletion, and cirrhosis. Thirty *ABCB4* variants were identified, including 18 novel variants.

**Conclusion:**

*ABCB4* gene-related cholestatic liver diseases have a wide spectrum of clinical and genetic variations. Biallelic *ABCB4* mutation carriers tended to severe PFIC3, which mostly occurs in children; while *ABCB4* non-biallelic variants can lead to milder ICP, LACP, DILI or overlapping, mostly in adults. Thus, the *ABCB4* genotype has a specific correlation with the phenotype, but there are exceptions. Non-biallelic null mutations can cause severe diseases. The mechanisms underlying this genetic phenotype require further investigation.

**Graphical Abstract:**

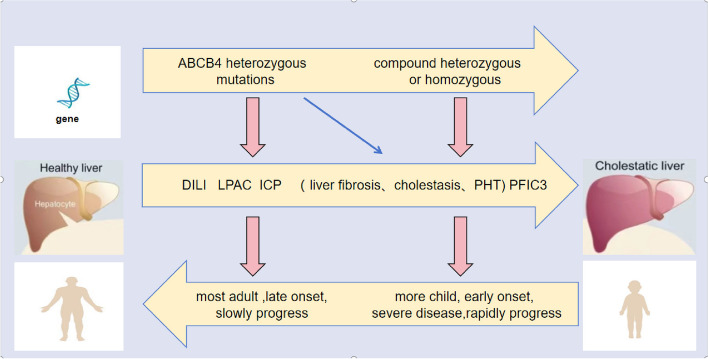

**Supplementary Information:**

The online version contains supplementary material available at 10.1186/s13023-024-03179-w.

## Background

Recently, with the in-depth study of the *ABCB4* (ATP binding cassette, sub-family B, member 4) gene, cholestatic liver diseases caused by *ABCB4* mutations have been increasingly recognized. *ABCB4* encodes Multi-Drug Resistant 3 (MDR3) protein, which is important in normal bile formation and lipid transport [1]. MDR3 is a phosphatidylcholine translocation enzyme response for emulsifying bile salts in the bile and preventing bile salt damage to the liver and bile duct epithelial cells [2]. Deficiency caused by mutations in *ABCB4* can lead to bile duct damage caused by bile salts, resulting in cholestasis and liver fibrosis. The outcome presents as a variety of cholestatic liver diseases, and even liver failure [3, 4]. Currently, more than 500 *ABCB4* mutations have been reported, most of which are missense mutations [5]. Cholestatic liver diseases related to *ABCB4* variants include progressive familial intrahepatic cholestasis type 3 (PFIC3), benign recurrent intrahepatic cholestasis, intrahepatic cholestasis of pregnancy (ICP), drug-induced liver injury (DILI), and low phospholipid associated cholelithiasis (LPAC) [1, 6]. Homozygous or compound heterozygous mutations in *ABCB4* are considered characteristic variants of PFIC3, whereas heterozygous mutations in single alleles have been reported in patients with LPAC and ICP [6]. However, the genotype-phenotype relationship underlying *ABCB4* gene-related cholestatic liver diseases still need further explored.

In this study, the clinical and pathological characteristics of 23 patients with *ABCB4*-related cholestatic liver diseases were exhibited. Their genotype and its correlation with the clinical phenotype were analyzed to offer insights into this disease and enrich the *ABCB4* mutation spectrum.

## Methods

### Patients

Twenty-three patients with cholestatic liver diseases related to *ABCB4* were included, including 15 children and 8 adults, were diagnosed in the Department of Hepatology, Fifth Medical Center, PLA General Hospital between January 2009 and December 2022. Only patients in whom with disease-causing variations in the *ABCB4* gene were identified were included in the study. In all patients, including 14 patients with biallelic variants and 9 patients with non-biallelic variants. The clinical and genetic data of the 23 patients were collected, including general information, clinical symptoms, biochemical examination, imaging, liver pathology, and genetic information. All records of return visits or telephone follow-ups of the 23 patients were collected and analyzed.

### Diagnostic criteria

Diagnosis was based on clinical manifestations and genetic examinations of the patients. Patients with cholestasis or liver fibrosis carrying biallelic *ABCB4* mutations (pathogenic, likely pathogenic, or variants of uncertain significance (VUS) predicted pathogenic) were diagnosed with PFIC3 [7]. Chronic cholestasis was considered in non-biallelic *ABCB4* mutation carrier when it persisted for > 6 months [8]. Cirrhosis was diagnosed according to the 2019 Guidelines for the Diagnosis and Treatment of Cirrhosis [9]. LPAC was diagnosed when the patient met at least two of the following criteria: biliary symptoms present at ≤40 years old, intrahepatic echo or small stones present after cholecystectomy, and the recurrence of biliary tract symptoms. Pregnant women with unexplained pruritus and elevated serum bile acid and gamma-glutamyl transferase (GGT) levels, which were relieved after delivery, were diagnosed with ICP. After excluding other causes of liver injury, DILI was diagnosed when there was a time relationship between drug exposure and the onset of liver disease symptoms, or when elevated liver enzymes were associated with improvement after drug withdrawal [10]. All patients were excluded from viral hepatitis, non-hepatophil toxic hepatitis, autoimmune liver disease, and non-alcoholic fatty liver disease.

All patients in this study were treated with ursodeoxycholic acid (UDCA) at the dose of 10 ~ 15 mg/kg/d. The response to treatment was considered complete if the liver serum markers returned to normal.

### Genetic testing and mutation analysis

This study was approved by the Ethics Committee of the Fifth Medical Center of PLA General Hospital (KY-2023-9-57-1). After the patients or their parents signed the informed consent, blood samples were sent to Beijing GrandOmics Biosciences Co, Ltd. A next-generation sequencing panel of liver diseases was used to determine genetic causes. Pathogenic variants were verified by Sanger sequencing of their parents. Several algorithms were used to predict the pathogenicity of the novel mutations. PyMOL was used to visualize the three-dimensional structure of the wild-type and mutant proteins. The pathogenicity of the mutation was classified according to the American College of Medical Genetics and Genomics (ACMG) guidelines. To study genotype-phenotype correlation, patient genotypes were classified as biallelic mutations and singe heterozygous mutation including null mutations).

## Statistical analysis

Data were analyzed using the IBM SPSS Statistics 19 software (IBM, Armonk, NY, USA). Normally distributed data were expressed as mean ± SD and then compared using a single sample t-test. Data with skewed distributions are presented as median values (P25, P75), and comparisons were conducted using the Mann–Whitney U-test. Statistical significance was set at *P* < 0.05.

## Results

### General information

Twenty-three Han Chinese patients were diagnosed with *ABCB4*-related cholestatic liver diseases from 21 families consisting of 15 children and 8 adults. The mean age of onset was 13 (0.2–37) years of age. Twenty-two patients had non-consanguineous parents, except for Patient 12, whose parents were cousins. The sister of patient 12 died at the age of 10 years due to “liver cirrhosis and gastrointestinal bleeding.” Patients 1 and 2 were brothers. Patients 15 and 23 had a father-son relationship. The remaining patients belonged to unrelated families.

### Clinical phenotype and biochemical results

The clinical features of the 23 patients are shown in Table S1. Among the 15 pediatric patients, 11 with biallelic *ABCB4 *mutation were diagnosed with PFIC3; four patients with non-biallelic mutations were diagnosed as DILI, cirrhosis cholestasis, cirrhosis, and mild liver fibrosis respectively. Among the 8 adult patients, two with biallelic mutation phenotype were diagnosed with PFIC3; one with biallelic mutation (including one benign mutation) presented with ICP cirrhosis; five cases with non-biallelic mutations, two cases presented with ICP cirrhosis (overlapping DILI), and three cases were diagnosed with LPAC. The imaging findings of all patients (abdominal B-ultrasound/CT/MRI) indicated liver fibrosis. The initial complaints of the 23 patients were liver and spleen enlargement (4/23, 17.4%), abdominal distension (2/23, 15.4%), jaundice (8/23, 34.8%), and abnormal liver function without any symptoms (9/23, 39.1%).

At initial diagnosis, GGT was increased in all patients (262 ± 168) U/L, alanine transaminase in 18 cases (78.3%), aspartate aminotransferase in 21 cases (91.3%), total bilirubin in 16 cases (69.6%), direct bilirubin in 17 cases (73.9%), and total bile acid in 20 cases (87.0%). Fifteen patients (65.2%) presented with cholestasis, of whom 10 (43.5%) presented with chronic cholestasis (Table S1).

### Pathological characteristics

Liver histopathology was performed in 19 (82.6%) of the 23 patients, including 11 (77.3%, 11/15) children and 8 (100%, 8/8) adults. Four children did not undergo hepatocentesis because of decompensation of cirrhosis.

Pathological characteristics of the 19 patients included cystic fibrosis (100%), positive CK7 expression (89.5%), small bile duct hyperplasia (84.2%), bile duct deletion (5.3%), positive copper staining (42.1%), and cirrhosis (63.2%). There were no significant differences in the pathological changes between children and adults (Table 1).

**Table 1:** Characteristics of liver histopathology and immunohistochemical staining in 23 patients with *ABCB4* gene-related cholestatic liver disease

Group	Patient NO	Bile duct injury	Fibrosis of the sink area	Megakaryocyte	Grade (G) Fibrosis (S)	CK7	CU
Child	1	Small bile duct hyperplasia	YES	NO	G2S4	YES	+
	2	Small bile duct hyperplasia	YES	NO	G2S4	YES	+
	3	No obvious abnormalities in the small bile duct	NO	YES	G1–2S1–2	NO	–
		Interlobular bile duct stenosis and small bile duct hyperplasia	YES	NO	G2S4	YES	+
	4	Interlobular bile duct hyperplasia, lumen irregular narrowing, bile duct epithelial atrophy, vacuolation, loss	YES	NO	G2S2	YES	–
	5	Edema of the interlobular bile duct epithelium, hyperplasia of the bile duct with small vacuoles	YES	NO	G1–2S4	YES	–
		Stenosis of the interlobular bile duct and hyperplasia of the small bile duct	YES	NO	G2S2	YES	–
	6	Small bile duct hyperplasia	YES	NO	G2S3	YES	–
	7	No obvious abnormalities in the small bile duct	YES	NO	G1–2S4	NO	–
	8	Interlobular bile duct lumen stenosis and occlusion, bile duct epithelialization vacuolation, small bile duct hyperplasia	YES	NO	G2S2	YES	–
	9	NA					
	10	NA					
	11	Biliary lumen stenosis and atresia were accompanied by fibrosis and sclerosis. The small biliary duct hyperplasia was obvious and eroded the boundary plate	YES	NO	G2S4	YES	++
	12	NA					
	13	The lumen of the interlobular bile duct is irregular and narrow, and the small bile duct is hyperplasia	YES	NO	G2S4	YES	+
	14	NA					
	15	No obvious abnormalities in the small bile duct	YES	NO	G1S1	NO	–
Adult	16	Small bile duct hyperplasia	YES	NO	G1S4	YES	–
	17	Small bile duct hyperplasia	YES	NO	G2S4	YES	+
	18	NA	YES	NO	G2S4	NA	NA
		Small bile duct hyperplasia	YES	NO	G1S2	YES	+
	19	Reduced interlobular bile duct, bile duct epithelium injury, small bile duct hyperplasia	YES	NO	G2S1	YES	–
		The epithelium of the interlobular bile duct was atrophic and lost, typical interlobular bile duct was rare, and small bile duct hyperplasia was obvious	YES	NO	G2S2	YES	–
	20	Small bile duct hyperplasia	YES	NO	G2S4	YES	–
	21	Small bile duct hyperplasia	YES	NO	G3S4	YES	–
	22	Ductopenia	YES	NO	G1S3–4	YES	+
	23	The epithelium of the interlobular bile duct is atrophic and absent, and the small bile duct is hyperplasia	YES	NO	G1S2	YES	–

*NA* not available, − negative, + positive, ++ Strongly positive, *CU* copper staining

**Fig. 1:**
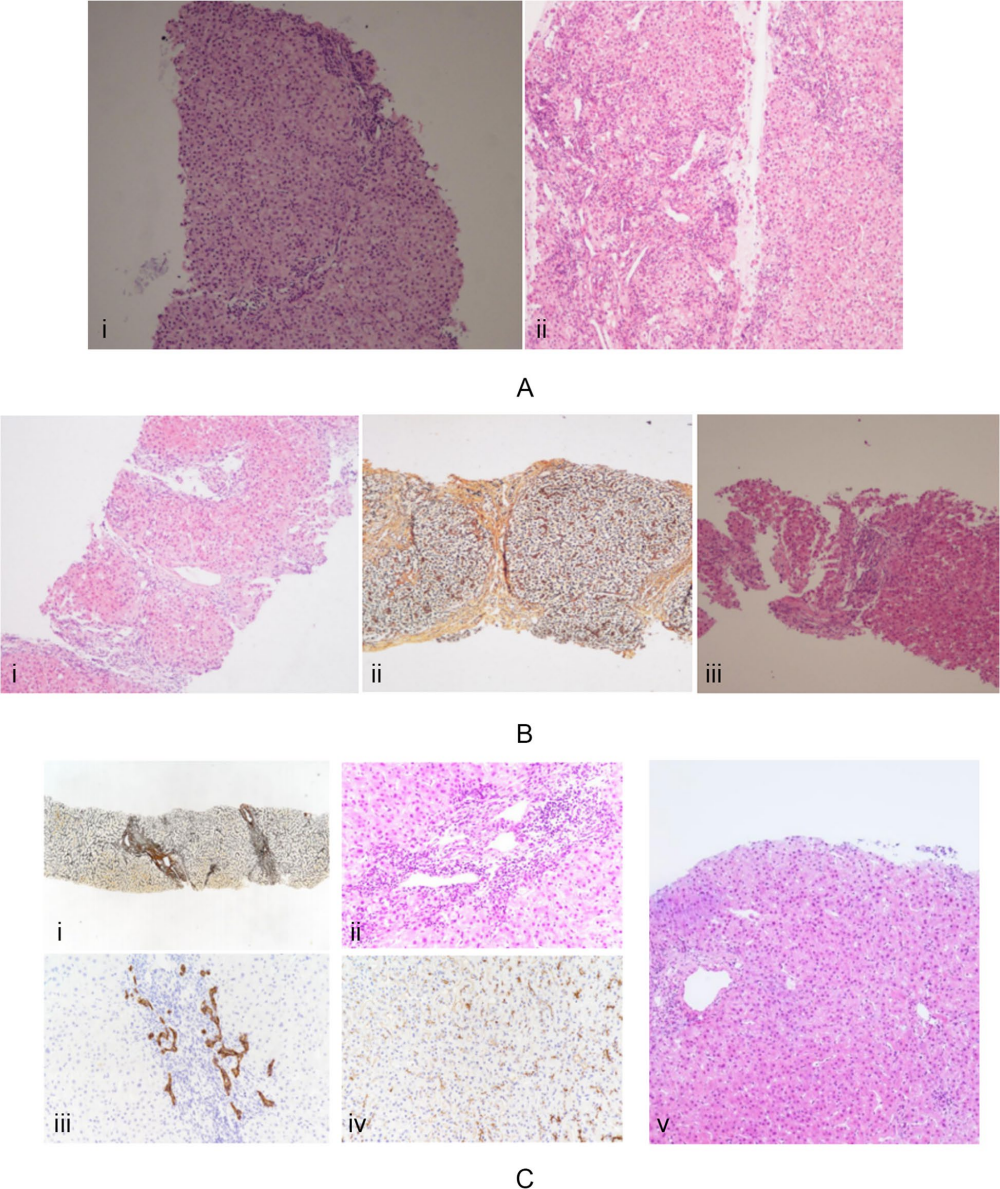
Histological findings of liver biopsies in patients underwent twice liver punctures. **A** Patient 3: (i) first hepatic histopathology: G1–2S1–2, no significant abnormalities in the small bile duct; (ii) second hepatic histopathology: G2S4, interlobular bile duct stenosis, small bile duct hyperplasia. **B** Patient 5: (i-ii) first hepatic histopathology: G1–2S4, pseudolobular structure formation, interlobular bile duct epithelial edema, vacuolation, and small bile duct hyperplasia; (iii) second hepatic histopathology: G2S2, interlobular bile duct stenosis, and small bile duct hyperplasia. **C** Patient 19: (i-iv) first hepatic histopathology: (i) lobular structure disorder (rete); (ii) bile duct absence, CK7+, chronic bile salt stasis; (iii) mild interstitial fibrosis in the junction area; (iv) spot-like necrosis, decreased CD10 expression in some areas (vanishing cholangiography syndrome, biliary liver fibrosis, G2S1); (v) second hepatic histopathology: atrophy and loss of interlobular bile duct epithelium, typical interlobular bile duct is rare in some convergence areas, small bile duct hyperplasia is obvious, mild interlobular inflammation (G2S2)

Patient 3, 5 18, and 19 underwent the hepatic histopathology twice. Patient 3 underwent the hepatic histopathology at 9 months and 2 years without UDCA treatment during this period (Fig. 1A), which was aggravated. Patient 5 underwent the hepatic histopathological examination at 4 years or 7 years, with continuous UDCA treatment in this period, showed remission (Fig. 1B).

Patient 18 showed G2S4 in the first hepatic histopathology (28 years old). After continued UDCA treatment, the second hepatic histopathology presented as G1S2 (32 years of age), indicating that the disease was alleviated. Patient 19 underwent the hepatic histopathology at 23 years and 30 years, indicating increased hepatic fibrosis (Fig. 1C).

**Fig. 2:**
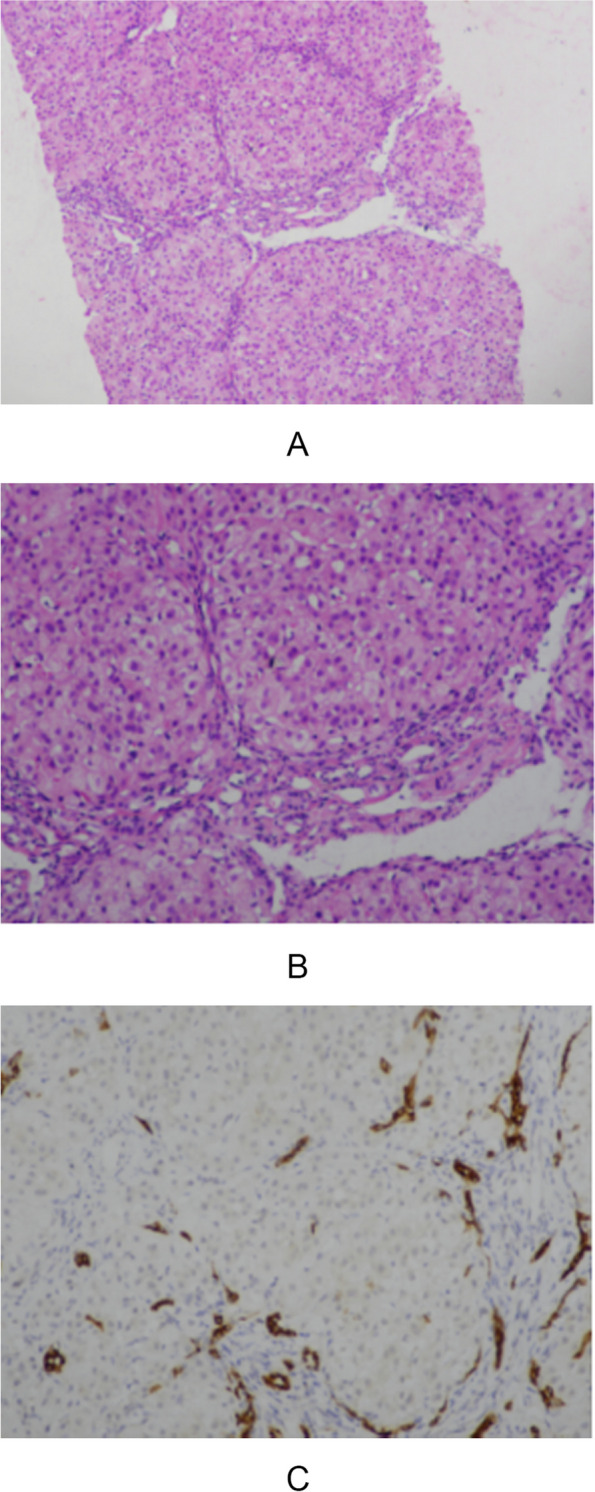
Histological findings of liver biopsy in representative patients with early onset of disease (patient 1). **A** G2S4, the hepatic lobule structure is disturbed, and false lobules have formed; (**B**) enlarged manifold area, fibrous tissue hyperplasia, interlobular bile duct hyperplasia; (**C**) numerous inflammatory cells infiltrated, mild fine bile duct reaction (CK7+)

The pathological features patient 1 (10 months old) was shown in Fig. 2, indicating the disease is serious.

### Gene mutation analysis

In this study, 30 *ABCB4* gene mutations were detected in 23 patients, including 20 missense mutations, five small deletions (< 21 bp), four splicing mutations, and one nonsense mutation (Table S2). The frameshift, nonsense, canonical ±1 or 2 splicing-site variants and exon deletions were defined as null variants, while missense and non-canonical splicing sites, as non-null variants, according to the ACMG standards [11].

**Fig. 3:**
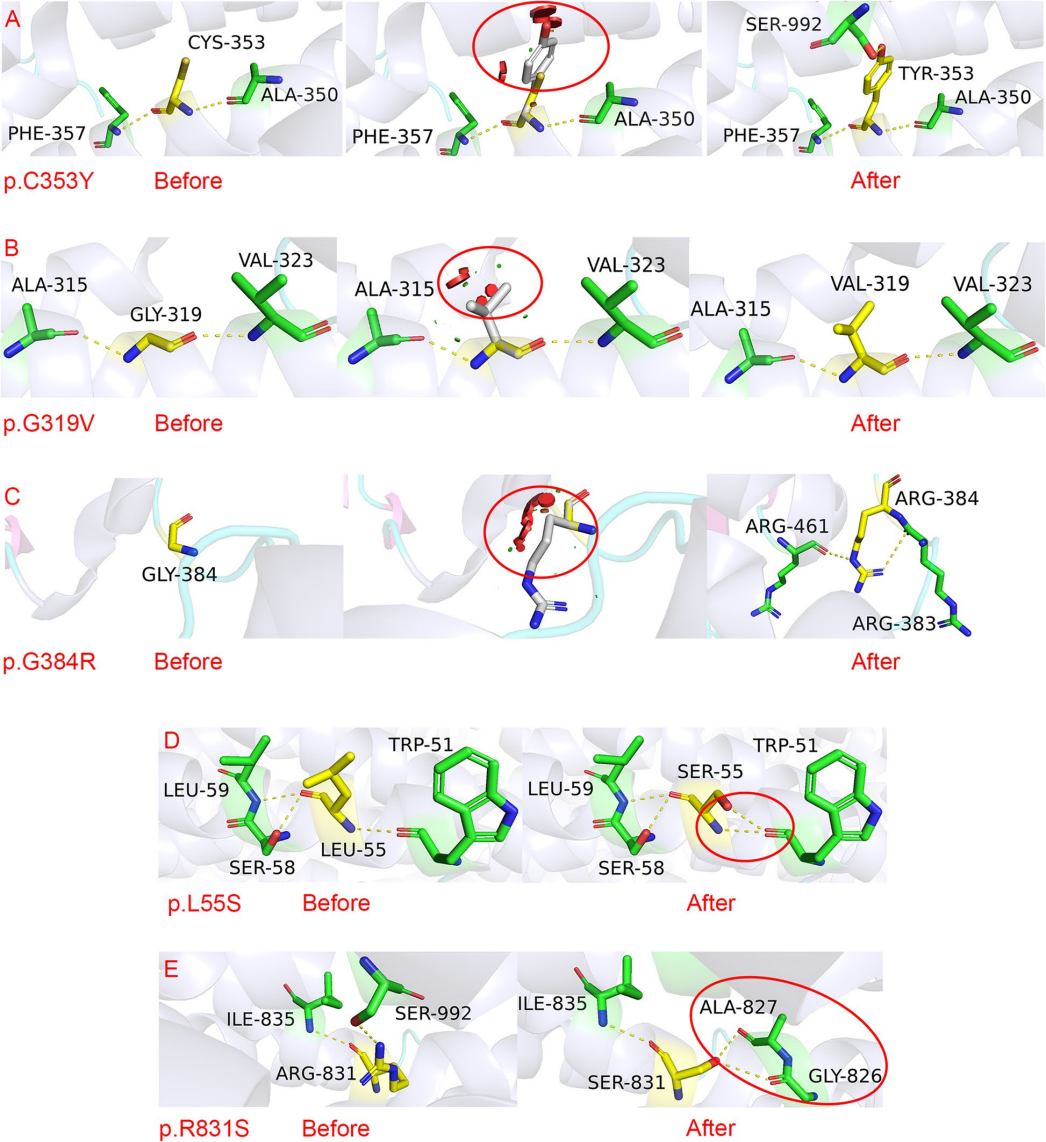
Novel missense mutations that predict changes in crystal structure. The mutation sites and surrounding residues are shown in yellow and green sticks, respectively. **A** p.C353Y: predicted to produce polar interaction with SER992. **B** p.G319V: predicted to produce repulsion. **C** p.G384R: predicted to produce new polar interactions with ARG461, ARG384, and ARG383. **D** p.L55S: predicted to produce a new polar interaction with TRP51. **E** p.R831S: the polar interaction with SER992 disappears, and polar interactions with ALA827 and GLY826 are generated

**Fig. 4:**
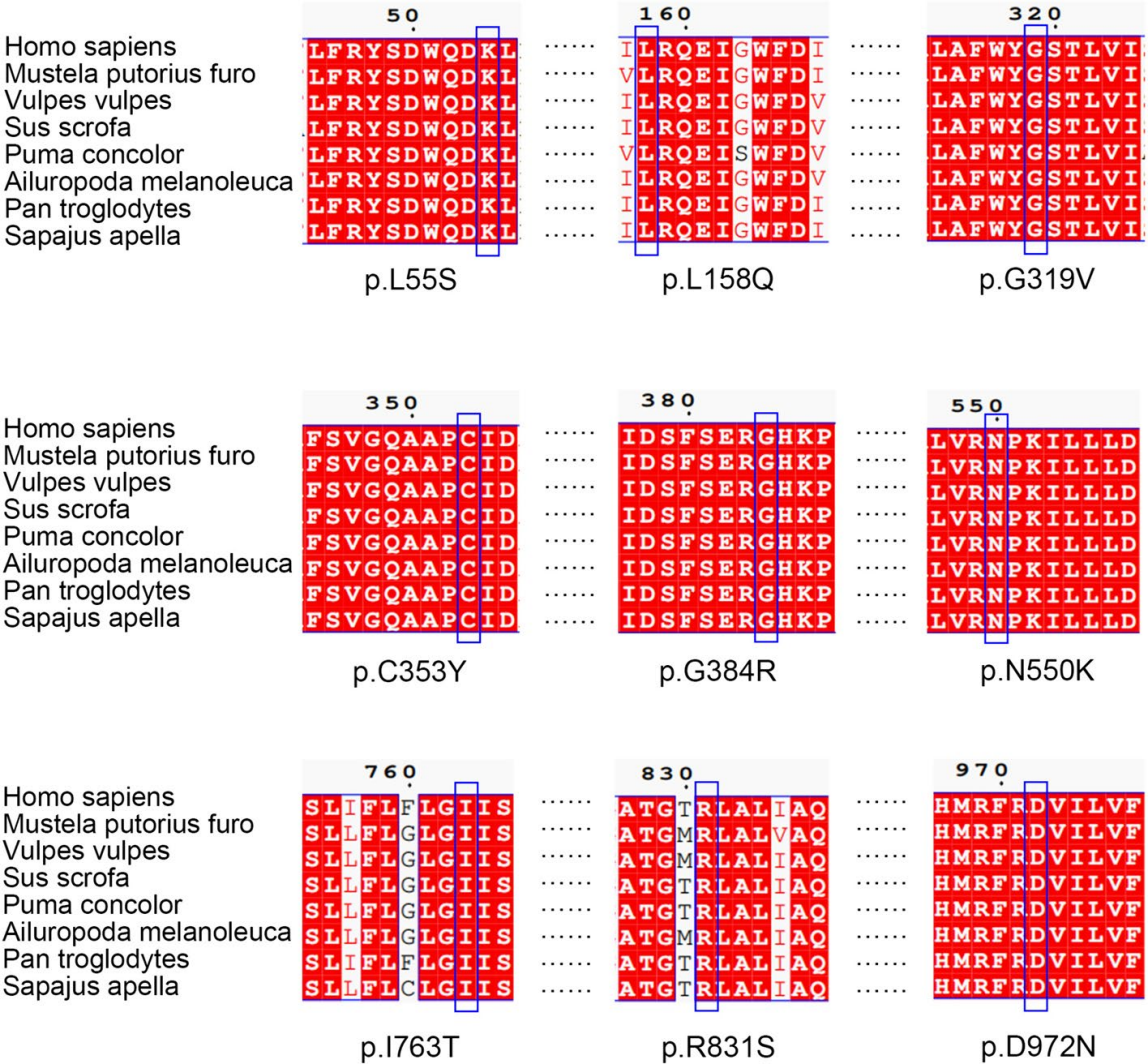
Conservation analysis of the nine missense variants among different species. Multispecies alignments of ABCB4 protein are shown. The mutation sites are labeled by blue rectangle

Eighteen mutation sites were novel, whereas the remaining sites were reported as disease-causing mutations in the Human Gene Mutation Database. Except for c.2394 + 82C > T, the other VUS were predicted to be pathogenic using the in silico algorithm (Table 2). For novel missense mutations, PyMOL was used to visualize the three-dimensional structure of the ABCB4 protein, showing the impact of the variants on the protein structure (Fig. 3, Fig. S1). The multispecies conservative prediction of novel missense mutations indicated that the mutation sites were highly conserved (Fig. 4). In this case, the mutation may have affected the protein function.

**Table 2:** Bioinformatics analysis of 18 novel *ABCB4* gene variants

Mutations	MutationTaster	PolyPhen-2	FATHMM-MKL	CADD	Varseak	SpliceAI	SIFT	FATHMM-indel	3D protein model
c.2949del (p.A984Qfs*4)	D	–	–	–	–	–	D	D	–
c.589C > T (p.Q197*)	D	–	D	D	–	–	–	–	–
c.1230 + 1G > A	–	–	D	D	S (CLASS 5: splicing effect)	S (Donor Loss score: 0.91; Donor Gain score: 0.38)	–	–	–
c.2914G > A (p.D972N)	D	B	D	D	–	–	–	–	YES
c.33–48delCTGGCGCCCCACGAGC (p.W12Rfs*21)	D	–	–	–	–	–	N	N	–
c.3252_3264del p.F1085Wfs*57	D	–	–	–	–	–	D	–	–
c.1058G > A (p.C353Y)	D	P	B	D	–	–	–	–	YES
c.956G > T (p.G319V)	D	ProbD	D	D	–	–	–	–	YES
c.473 T>A (p.L158Q)	D	ProbD	D	D	–	–	–	–	YES
c.164 T>C (p.L55S)	D	ProbD	D	D	–	–	–	–	YES
c.2288 T > C (p.I763T)	D	ProbD	D	D	–	–	–	–	YES
c.2493G > C (p.R831S)	D	ProbD	D	D	–	–	–	–	YES
c.1150G > C (p.G384R)	D	ProbD	D	D	–	–	–	–	YES
c.1650C > A (p.N550K)	D	PossD	D	D	–	–	–	–	YES
c.2317-5A > G (splice)	–	–	B	D	S (CLASS 5: splicing effect)	S (Acceptor Loss score: 0.55; Acceptor Gain score: 0.54)	–	–	–
c.2478 + 6 T > C (splice)	–	–	D	D	S (CLASS 4: Likely splicing effect)	S (Donor Loss SCORE: 0.76)	–	–	–
c.3269_3271del (p.Ala1090del)	D	–	–	–	–	–	D	D	–
c.2394 + 82C > T	–	–	B	B	N	N	–	–	–

*D* disease causing/deleterious, − not applicable, *B* benign, *P* polymorphism, *ProbD* probably damaging, *PossD* possibly damaging, *S* splicing potential alteration of splicing, *N* Neutral

### Analysis of association between phenotype and genotype

In this study, 11 children and two adults with biallelic *ABCB4* mutations presented the phenotype of PFIC3. One adult with compound heterozygous mutations presented with ICP cirrhosis caused by a benign mutation. Four children and five adults with non-biallelic *ABCB4* mutations were phenotypically diverse, ranging from DILI, ICP, and LPAC, to liver transplantation due to severe liver fibrosis (one child). In conclusion, patients with biallelic mutations in *ABCB4* have a young age of onset and rapid disease progression. Their clinical phenotypes were mostly consistent with that of PFIC3, which is a common phenotype in children. The diseases in non-biallelic mutation carriers are relatively mild and late onset and are significant in adults. Although the clinical phenotype of most *ABCB4* mutation carriers conforms to the genotype-phenotype relationship mentioned above, there are exceptions. The age of onset in Patient 9 (child), who carried a heterozygous variant (c.2525 T > C, p.L842P), was 6 years. His clinical phenotypes included cirrhosis and cholestasis, which progressed rapidly. He underwent liver transplantation for end-stage liver disease at the age of 12 years.

In the present study, biallelic mutations played a dominant role in the children and non-null variants (missense) were more. Non-biallelic mutations were predominant in adults, and most of them were null various. The types of gene mutations were diverse, and the clinical phenotypes of deletion mutations were relatively severe.

### Treatment follow-up and prognosis

All patients with *ABCB4*-related cholestatic liver diseases were treated with UDCA, glycyrrhein, glutathione, and butyldisulfonate methionine during hospitalization. Except for Patient 3, 9, 17, and 20, all patients responded partially or fully to UDCA treatment. The follow-up time was 6.7 (0.5–13.5) years. One adult patient (Patient 20) died of liver failure while four children underwent liver transplantation. Cirrhosis continued to progress in six patients (four children and two adults). The conditions of the remaining 12 patients remained stable.

## Discussion

MDR3 is encoded by *ABCB4* and expressed on the tubule membranes of hepatocytes. It is responsible for secreting phospholipids (mainly phosphatidylcholine) into bile [12]. Choline neutralizes the washing action of hydrophobic bile salts by forming mixed micelles. Therefore, MDR3 deficiency leads to intrahepatic cholestasis, resulting in damage to the bile duct epithelium and membranes [13, 14]. In addition, low phospholipid levels lead to micellar instability, which promotes cholesterol crystallization and increases biliary lithiasis. The elevated lithiasis promotes liver damage by blocking the small bile ducts, resulting in cholestasis characterized by high GGT levels. Pathogenic biallelic mutations of *ABCB4* can lead to a disease spectrum from the PFIC3, to LPAC, ICP, or DILI, Which are less severe. However, a clear association of clinical phenotype and genotype is lacking due to the complexity and overlap of clinical symptoms. Studies on the genotypic-phenotypic correlation of *ABCB4*-related cholestatic liver diseases [15–18] suggested that those with compound heterozygous or homozygous *ABCB4* pathogenic mutations have a higher proportion of progression to cirrhosis and end-stage liver disease, whereas those with heterozygous mutations usually have mild lesions or even no symptoms [19].

**Fig. 5:**
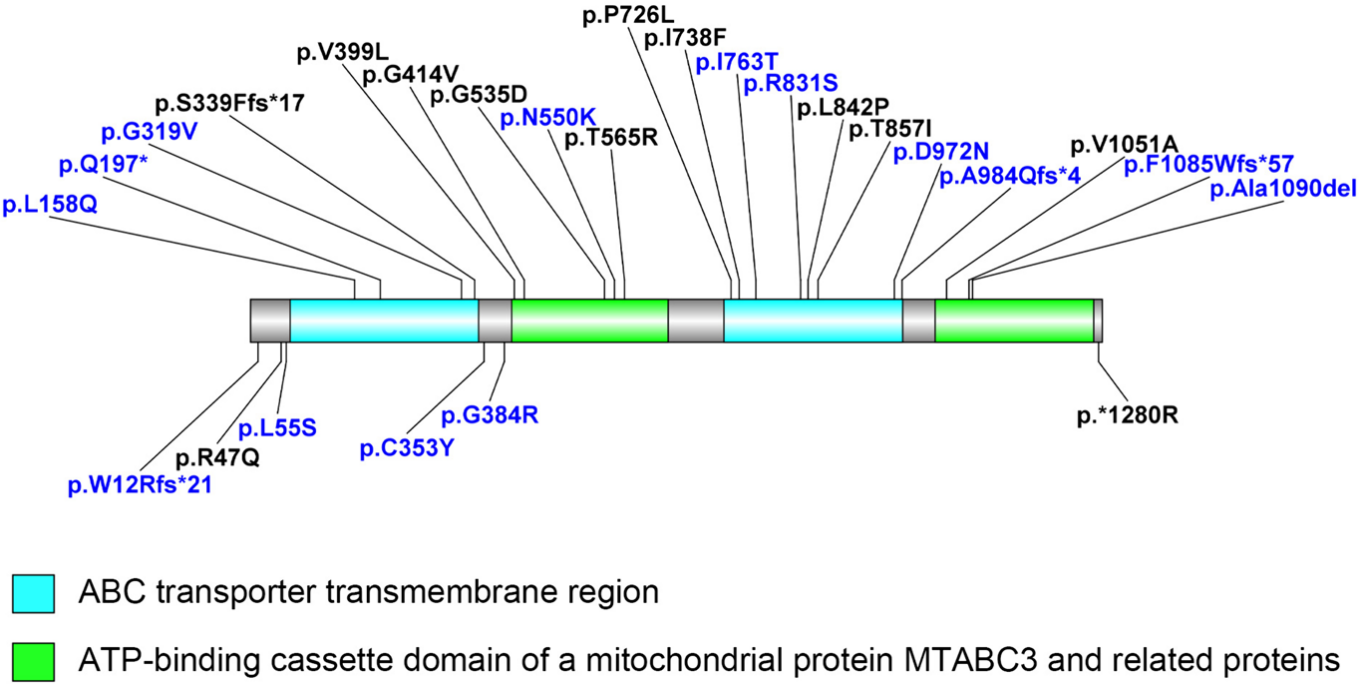
The diagram of the ABCB4 protein. The *ABCB4* variants position in our cohort. The reported variants are shown in black, while the novel variants are in blue

This study describes 23 patients with *ABCB4* mutations from 21 unrelated families, including 11 children and two adults with biallelic mutations, diagnosed with PFIC3. Thirty mutations were identified, of which 18 were novel. Their positions on the MDR3 protein are shown (Fig. 5). The MDR3 protein is composed of 1286 amino acids, organized into two repeats. Each repeat contains approximately 610 amino acids and joined by a 60-residue linker region. A transmembrane domain (TMD) and a cytoplasmatic nucleotide-binding domain (NBD) are identified within each repeat. The TMD located on the extracellular side and was composed of six transmembrane α-helices, containing specific sites for substrate, such as phosphatidylcholine of the phospholipid family. While the NBD is on the cytoplasmatic side of the protein, obtaining the energy obtained from ATP hydrolysis for substrate transport. The TMD and NBD jointly exert the function of the MDR3 protein. Mutations located in two domains may cause changes in the structure and function changes of the MRD3 protein.

PFIC3 is associated with biallelic mutations of *ABCB4*, clinically characterized by cholestasis with jaundice and pruritus. The onset symptoms can appear in late infancy, adolescence, and even young adulthood, and progress to cirrhosis and end-stage liver disease. In this cohort, 13 patients were diagnosed with PFIC3 at a minimum age of 2 months. Ten children developed either cirrhosis or decompensation. Two adults had progressive cirrhosis and one died of liver failure. Relatively mild clinical phenotypes such as LPAC, ICP, and DILI are related to non-biallelic mutations of *ABCB4* that retain at least part of the protein function [6]. Of the 11 cases that carried compound heterozygous mutations, one carrier of nonsense variant and two carrier of deletion or frameshift variants, presenting severe clinical symptoms. Among them, one adult patient (c.3269_3271del and c.3252_3264del) died of end-stage liver disease at 24 years of age. Two children who underwent liver transplantation due to progression to end-stage liver disease had frameshift and splice mutations. While a 38-year-old female patient with compound heterozygous mutations, including a benign mutation and a frameshift mutation, presented with ICP cirrhosis, although the disease had a relatively late onset. Therefore, mutations that cause truncated or absent ABCB4 proteins, such as nonsense, frameshift, and splice mutations, can lead to more severe clinical phenotypes than missense mutations.

In this study, one child with a homozygous mutation (c.1241G > T) was diagnosed with cirrhosis at 2 years of age, indicating the high pathogenicity of homozygous *ABCB4* mutations. One adult heterozygous carrier of the same variant (c.1241G > T), presented with LPAC, suggesting that homozygous *ABCB4* mutations can cause earlier onset age and a more severe phenotype. Two children homozygous for the same mutation site (c.1195G > C) had onset ages of 3 and 13 years, respectively. The onset phenotype was severe decompensated cirrhosis [20]. However, three siblings were reported to carry a homozygous mutation site and exhibited mild phenotypes. A possible explanation is that multiple factors, including hormones, the environment, and other genetic factors, contribute to the occurrence of *ABCB4* deficiency-related liver diseases.

Nine patients carried non-biallelic *ABCB4* mutations in our cohort. ICP usually occurs in the middle and third trimesters of pregnancy [21]. The main clinical phenotypes include maternal pruritus and elevated serum bile acid concentration [22], which rapidly resolve after delivery. LPAC is a rare type of intrahepatic cholelithiasis that often occurs in young adults [17]. The use of drugs that may inhibit ABCB4 protein function or expression can lead to DILI [23]. These relatively mild clinical phenotypes may still progress to liver fibrosis during the course of the disease. The late onset mild phenotype related to non-biallelic mutations may be related to partially retained protein function [24]. The splice and deletion mutation carriers showed a relatively severe phenotype than the missense mutation carriers in non-biallelic mutation carriers group, indicating that the null variants were found to be more pathogenic. However, the heterozygous missense mutations were also identified in children which means they are related to the early onset. The possible reason for heterozygous missense mutations leading to early onset age may attributed to their location in the ABC transporter transmembrane region, which can provide specificity for the substrate (Fig. 5). In addition, there is one except that heterozygous missense mutations carrier (c.2525 T > C p.L842P) developed end-stage liver disease and was treated with liver transplantation. This patient had no history of medication use or aggravated induction. A patient with compound heterozygous variants (p.L842P and p.V1051A) was reported to present with the phenotype of PFIC3 [25]. The mutation p.L842P was verified to come from the mother who had gallstones and underwent cholecystectomy, whereas the mutation p.V1051A came from the normal father. Thus, the mutation p.L842P is considered to have a greater effect on the ABCB4 protein function. However, the mother of our patient who carried p.L842P showed no obvious clinical symptoms. The different phenotypes caused by the same mutation may be associated with epigenetic factors, which require further investigation.

The liver histopathology of cholestatic liver disease associated with *ABCB4* mainly involves bile duct changes such as epithelial damage, atrophy, and cholesterol lysis. The two prominent pathological features of PFIC3 are portal fibrosis and bile duct hyperplasia, occasionally accompanied by mild giant cell hepatitis. Portal fibrosis and biliary cirrhosis are common in advanced stages. Liver histopathology of the 19 patients in our consistent with the previous study.

UDCA is the most common medication for cholestatic liver diseases patients. UDCA competes for the reabsorption of primary bile acids in the small intestine to promote their excretion, thus reducing the damage caused by cholestasis in hepatocytes [26]. Its immunomodulatory and anti-inflammatory properties have also been approved [27]. The serum transaminase levels and pruritus in more than half of the patients with *ABCB4* pathogenic mutations were proven to be effectively reduced by UDCA [21]. In our study, all patients were treated with UDCA and 12 (52.2%) achieved significant therapeutic effects. Four patients showed no response to UDCA, in which Patients 3 and 20 were diagnosed with PFIC3. Patient 3 carries two missense variants while Patient 20 carries one small in-frame deletion and one frameshift variant, which was consistent with research from France. However, one patient in our cohort was inconsistent with one of the conclusions of this study, which is that patients carrying two mutations that altered the start codon or modifying splicing showed no response to UDCA treatment. Patient 22 carries one frameshift and one splicing variant responded fully to UDCA treatment [28]. We will conduct continuous follow-ups on him in the future to further investigate the possible causes of the difference. Patients who showed no clear response were considered clinically serious (cirrhosis with portal hypertension). Three patients who received long-term UDCA treatment showed lesion remission in the second liver puncture. In contrast, the youngest child without lesions in the first pathological examination was not regularly treated with UDCA. Liver pathology in a one-year review indicated that the pathology of liver fibrosis was significantly aggravated. Therefore, whether UDCA alters the natural history of *ABCB4*-related cholestatic liver diseases remains unclear. However, the use of UDCA in the early stages may prevent acute and chronic complications and improve prognosis.

Liver transplantation is the last choice of treatment. Over a short follow-up period of 3–5 years, liver transplantation improved cholestasis-related symptoms in more than 75% of patients with PIFC3 [29, 30]. In this study, liver transplantation was performed on three patients at 10, 12, and 20 years old respectively. The patients were followed-up for 5, 1, and 4 years, showing stable conditions.

*ABCB4* variants may lead to different clinical phenotypes. When transaminase abnormalities with elevated GGT levels, liver fibrosis, and cholestasis are identified clinically in a patient, *ABCB4*-related liver diseases should be considered. Genetic testing are necessary for the final diagnosis to avoid misdiagnosis and delayed treatment. The early use of UDCA may improve the prognosis of *ABCB4*-related diseases. This study has a small sample size and limited data acquisition, which may have biases and certain limitations. Therefore, it is necessary to expand the sample size to further observe the diseases.

## Conclusions

This study identified 18 novel *ABCB4* mutations. The discovery and evaluation of these novel mutations enrich the spectrum of pathogenic mutations in *ABCB4* and can be used for future evaluation of patients. A certain correspondence between *ABCB4* genotype and clinical phenotype has been identified: Biallelic *ABCB4* mutation carriers tended to severe PFIC3, which mostly occurs in patients under 18 years old; while *ABCB4* non-biallelic variants can lead to milder ICP, LACP, DILI or overlapping, mostly in adults. However, Non-biallelic null variants also can cause severe diseases. The mechanism and genotype-phenotype correlation of *ABCB4* gene-related diseases need to be further explored.

### Supplementary Information

**Supplementary Material 1.**

## Data Availability

The data presented in this study are available on request from the corresponding author. The data are not publicly available due to restrictions privacy and ethical.
