# Supplementary material for: Clinical and genetic study of ABCB4 gene-related cholestatic liver disease in China: children and adults
Source: Orphanet J Rare Dis. 2024 Apr 12;19:157. doi: 10.1186/s13023-024-03179-w (PMC11010299; doi:10.1186/s13023-024-03179-w)
Supplement: Supplementary file 1 — Supplementary Material 1. [file 13023_2024_3179_MOESM1_ESM.docx]

**Supplementary Table 1. Clinical data and follow-up results of 23 patients with *ABCB4* gene-related cholestatic liver diseases.**

| Group | Patient NO | Gender | Age(Y) | ALT | AST | TBIL | DBIL | GGT | ALP | TBA | Imaging | Diagnosis | Treatment | Follow-up time(Y) | Ending |
| --- | --- | --- | --- | --- | --- | --- | --- | --- | --- | --- | --- | --- | --- | --- | --- |
| 0-18 years old | 1 | M | 0.7 | 111 | 127 | 10.6 | 6.3 | 126 | 411 | 124 | Hepatomegaly, cirrhosis | PFIC3 | UDCA | 7.4 | Stable |
|  | 2 | M | 2.3 | 104 | 190 | 37.6 | 22.4 | 112 | 337 | 30 | Cirrhosis, splenomegaly | PFIC3 | UDCA | 13.5 | Progress |
|  | 3 | F | 0.2 | 114 | 201 | 57.2 | 42.5 | 159 | 273 | 260 | Cirrhosis, splenomegaly | PFIC3 | UDCA | 9.2 | Progress |
|  | 4 | M | 2.5 | 90 | 88 | 8.4 | 6.1 | 538 | 424 | 7 | Hepatomegaly, splenomegaly, liver fibrosis | PFIC3 | UCDA | 5.3 | Stable |
|  | 5 | M | 3.11 | 142 | 121 | 30.6 | 23.8 | 362 | 419 | 118 | Hepatomegaly, splenomegaly | PFIC3 | UDCA | 7.5 | Stable |
|  | 6 | M | 3.8 | 138 | 172 | 29.6 | 15.8 | 427 | 467 | 19 | Cirrhosis, splenomegaly | PFIC3 | UDCA | 10.2 | Liver transplantation |
|  | 7 | F | 5.1 | 50 | 112 | 24.7 | 12.1 | 90 | 322 | 98 | Cirrhosis, splenomegaly | PFIC3 | UDCA | 6.2 | Stable |
|  | 8 | M | 6.10 | 221 | 149 | 211.7 | 125.3 | 411 | 1483 | 479.4 | Liver echo thickening | DILI | UDCA | 3.8 | Stable |
|  | 9 | M | 6.11 | 169 | 210 | 37 | 16.3 | 664 | 359 | 120 | Cirrhosis, splenomegaly | Cirrhosis, cholestasis | UDCA | 5.5 | Liver transplantation |
|  | 10 | M | 12.8 | 79 | 107 | 36.6 | 20.6 | 260 | 587 | 46 | Cirrhosis, splenomegaly | PFIC3 | UDCA | 5.4 | Liver transplantation |
|  | 11 | M | 12.2 | 203 | 214 | 80 | 62.5 | 158 | 340 | 147 | Cirrhosis, splenomegaly | PFIC3 | UDCA | 6.7 | Progress |
|  | 12 | F | 13 | 125 | 187 | 97.4 | 77 | 280 | 381 | 248 | Cirrhosis, splenomegaly | PFIC3 | UDCA | 2.5 | Progress |
|  | 13 | F | 14 | 51 | 86 | 23.5 | 9.1 | 126 | 223 | 25 | Cirrhosis, splenomegaly | Cirrhosis | UDCA | 6.8 | Stable |
|  | 14 | M | 15.6 | 85 | 127 | 56.4 | 36.9 | 457 | 306 | 108 | Cirrhosis, splenomegaly | PFIC3 | UDCA | 0.7 | Liver transplantation |
|  | 15 | M | 17.2 | 68 | 32 | 6.9 | 3.4 | 62 | 103 | 1 | Splenomegaly | Mild hepatic fibrosis | UDCA | 7.0 | Stable |
| Over 18 years old | 16 | F | 20 | 50 | 60 | 25.2 | 17.9 | 209 | 225 | 86 | Cirrhosis, splenomegaly | PFIC3 | UCCA | 9.3 | Stable |
|  | 17 | F | 20 | 52 | 98 | 183 | 156.3 | 93 | 345 | 223 | Cirrhosis, splenomegaly | ICP cirrhosis(DILI) | UDCA | 11.5 | Progress |
|  | 18 | F | 22 | 112 | 116 | 30.6 | 18.3 | 325 | 415 | 11 | Cirrhosis, splenomegaly | ICP cirrhosis (DILI) | UDCA | 12.7 | Progress |
|  | 19 | M | 23 | 379 | 4211 | 67.1 | 47.8 | 1506 | 373 | 47.5 | Cholecystectomy, splenomegaly | LPAC | UDCA | 4.0 | Stable |
|  | 20 | F | 25 | 20 | 56 | 341.3 | 276.9 | 422 | 622 | 85.5 | Cirrhosis, splenomegaly | PFIC3 | UDCA | 0.5 | Death |
|  | 21 | M | 29 | 33 | 49 | 235.8 | 180.7 | 105 | 232 | 81 | Cirrhosis, cholecystectomy | LPAC | CDCA | 5.8 | Stable |
|  | 22 | F | 35 | 33 | 34 | 18.4 | 7.0 | 177 | 165 | 62 | Cirrhosis, splenomegaly | ICP cirrhosis | UDCA | 1.0 | Stable |
|  | 23 | M | 37 | 39 | 44 | 13.7 | 4.6 | 193 | 114 | 7 | Cholecystectomy | LPAC | UDCA | 12 | Stable |

Y, year; ALT, alanine aminotransferase; AST, aspartate aminotransferase; TBIL, total bilirubin; DBIL, direct bilirubin; GGT, Gamma-Glutamyl Transferase; ALP, Alkaline phosphatase; TBA, total bile acids; M, male; F, female; PFIC3, progressive familial intrahepatic cholestasis type 3; LPAC, low phospholipid associated cholelithiasis; ICP, intrahepatic cholestasis of pregnancy; DILI, drug-induced liver injury; UDCA, ursodeoxycholic acid.

Reference ranges: ALT (5-40U/L); AST (8-40U/L); TBIL (3.4-20.5umol/L); DBIL (0-6.8umol/L); GGT (11-50U/L); ALP (40-150U/L); TBA (0-10umol/L).

**Supplementary Table 2. Genetic results of *ABCB4* (NM_000443) variants in the study population.**

| Group | Patient NO | Location on *ABCB4* gene | Nucleotide variat | Peptide variant | Type | Inheritance | Status | Reported in HGMD | ACMG classification | Phenotype |
| --- | --- | --- | --- | --- | --- | --- | --- | --- | --- | --- |
| 0-18 years old | 1 | Exon21  Exon18  Exon14 | c.2570C>T  c.2212A>T  c.1694C>G | p.T857I  p.I738F  p.T565R | Missense  Missense  Missense | Mother  Father  Father | Compound heterozygous | DM  DM  DM | -  -  - | PFIC3 |
|  | 2 | Exon21  Exon18  Exon14 | c.2570C>T  c.2212A>T  c.1694C>G | p.T857I  p.I738F  p.T565R | Missense  Missense  Missense | Mother  Father  Father | Compound heterozygous | DM  DM  DM | -  -  - | PFIC3 |
|  | 3 | Exon12  Exon12 | c.1241G>T  c.1241G>T | p.G414V  p.G414V | Missense  Missense | Mother  Father | Homozygous | DM  DM | -  - | PFIC3 |
|  | 4 | Exon25  Exon7 | c.3152T>C  c.589C>T | p.V1051A  p.Q197X | Missense  Nonsense | Mother  Father | Compound heterozygous | DM  No | -  LP | PFIC3 |
|  | 5 | Exon10  Exon9 | c.1058G>A  c.956G>T | p.C353Y  p.G319V | Missense  Missense | Mother  Father | Compound heterozygous | No  No | VUS  VUS | PFIC3 |
|  | 6 | Exon11  Exon11 | c.1195G>C  c.1195G>C | p.V399L  p.V399L | Missense  Missense | Father  Mother | Homozygous | DM  DM | -  - | PFIC3 |
|  | 7 | Exon6  Exon4 | c.473T>A  c.164T>C | p.L158Q  p.L55S | Missense  Missense | Father  Mother | Compound heterozygous | No  No | VUS  VUS | PFIC3 |
|  | 8 | Exon18 | c.2288T>C | p.I763T | Missense | Mother | Single heterozygous | No | VUS | DILI |
|  | 9 | Exon21 | c.2525T>C | p.L842P | Missense | Mother | Single heterozygous | DM | - | Cirrhosis cholestasis |
|  | 10 | Exon21  Exon12 | c.2493G>C  c.1230+1G>A | p.R831S  - | Missense  Splicing | Mother  Father | Compound heterozygous | No  No | VUS  LP | PFIC3 |
|  | 11 | Exon4  Exon11 | c.140G>A  c.1150G>C | p.R47Q  p.G384R | Missense  Missense | Father  Mother | Compound heterozygous | DM  No | -  VUS | PFIC3 |
|  | 12 | Exon11 | c.1195G>C  c.1195G>C | p.V399L  p.V399L | Missense  Missense | Mother  Father | Homozygous | DM  DM | -  - | PFIC3 |
|  | 13 | Exon17 | c.2177C>T | p.P726L | Missense | Father | Single heterozygous | DM | - | Cirrhosis |
|  | 14 | Exon10  Exon23 | c.1015dup  c.2914G>A | p.S339Ffs*17  p.D972N | Deletion  Missense | Mother  Father | Compound heterozygous | DM  No | -  LP | PFIC3 |
|  | 15 | Exon28 | c.3838T>C | p.*1280R | Missense | Father | Single heterozygous | DM | - | Mild hepatic fibrosis |
| Over 18 years old | 16 | Intron18  Exon14 | c.2317-5A>G  c.1650C>A | -  p.N550K | Splicing  Missense | Mother  Father | Compound heterozygous | No  No | VUS  VUS | PFIC3 |
|  | 17 | Intron20 | c.2478+6T>C | - | Splicing | — | Single heterozygous | No | VUS | ICP cirrhosis(DILI) |
|  | 18 | Exon21 | c.33_48delCTGGCGCCCCACGAGC | p.W12Rfs*21 | Frameshift | Mother | Single heterozygous | No | LP | ICP cirrhosis (DILI) |
|  | 19 | Exon12 | c.1241G>T | p.G414V | Missense | Mother | Single heterozygous | DM | - | LPAC |
|  | 20 | Exon25  Exon25 | c.3269_3271del  c.3252_3264del | p.Ala1090del  p.F1085Wfs*57 | Deletion  Frameshift | Spontaneous  Spontaneous | Compound heterozygous | No  No | VUS  LP | PFIC3 |
|  | 21 | Exon14 | c.1604G>A | p.G535D | Missense | — | Single heterozygous | DM | - | LPAC |
|  | 22 | Exon24  Intron19 | c.2949del  c.2394+82C>T | p.A984Qfs*4  - | Frameshift  Splicing | Mother  father | Compound heterozygous | No  No | P  VUS | ICP cirrhosis |
|  | 23 | Exon28 | c.3838T>C | p.*1280R | Missense | — | Single heterozygous | DM | - | LPAC |

DM, disease-causing mutations; PFIC3, progressive familial intrahepatic cholestasis type 3; DILI, drug-induced liver injury; ICP, intrahepatic cholestasis of pregnancy; LPAC, low phospholipid associated cholelithiasis; LP, likely pathogenic; VUS, Variant of Uncertain Significance; P, pathogenic

**Supplementary Figure 1. Novel missense mutations that predict no changes in crystal structure.**


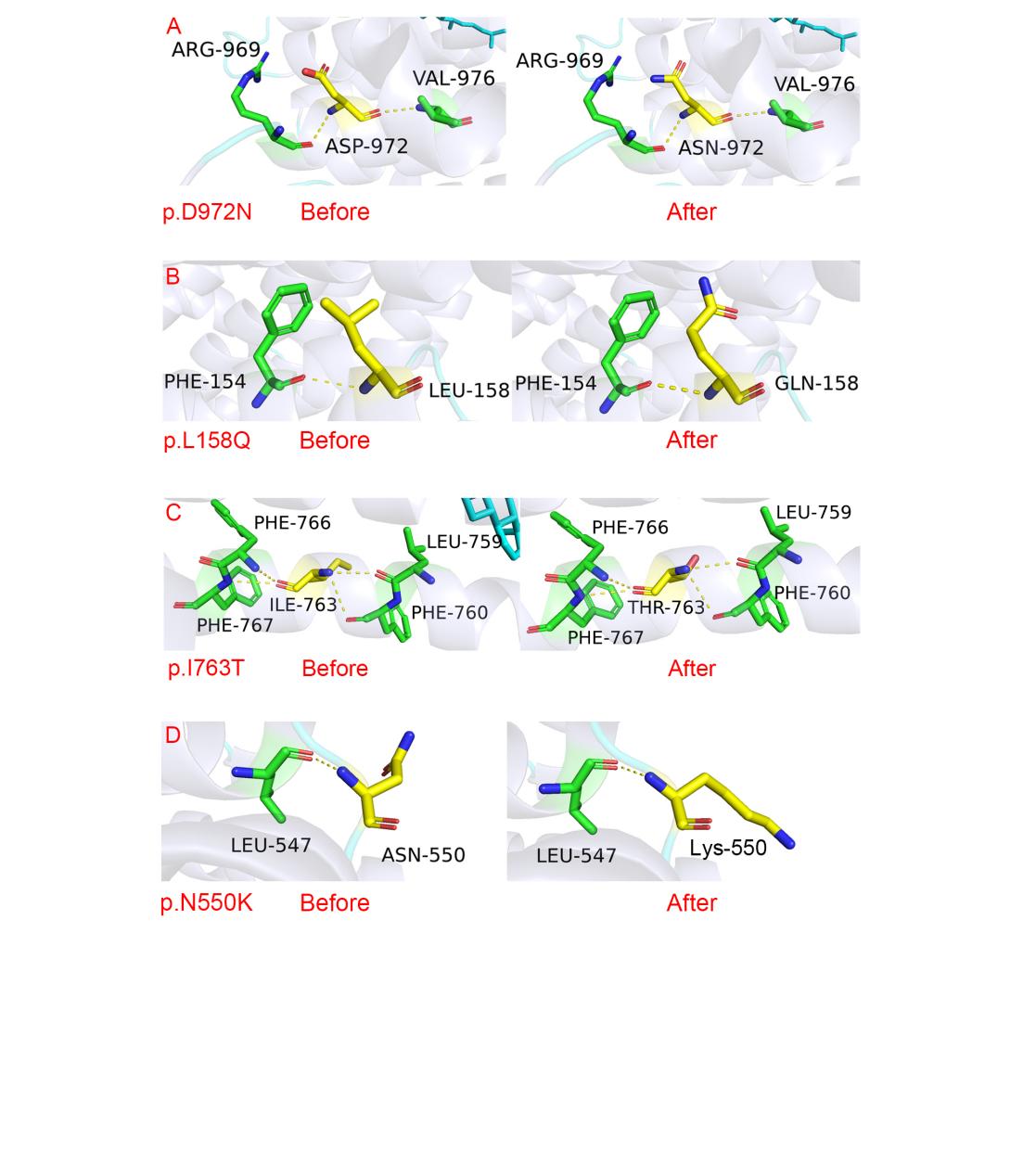


**Figure legend:** The mutation sites and surrounding residues are shown in yellow and green sticks, respectively. (A) ASP972 changed to ASN972, predicted no change in the polar interaction. (B) LEU158 changed to GLN158, predicted no change in the polar interaction. (C) ILE763 changed to THR763, predicted no change in the polar interaction. (D) ASN550 changed to LYS550, predicted no change in the polar interaction.
